# The role of the basal forebrain in general anesthesia

**DOI:** 10.1002/ibra.12082

**Published:** 2022-12-03

**Authors:** Yi‐Ting Peng, Cheng‐Dong Yuan, Yi Zhang

**Affiliations:** ^1^ Department of Anethesiology The Second Affiliated Hospital of Zunyi Medical University Zunyi Guizhou China; ^2^ Guizhou Key Laboratory of Anesthesia and Organ Protection Zunyi Medical University Zunyi Guizhou China; ^3^ School of Anesthesiology Zunyi Medical University Zunyi Guizhou China

**Keywords:** basal forebrain, consciousness, general anesthesia, neural pathway, neurons

## Abstract

The basal forebrain is a group of nerve nuclei on the ventral side of the ventral ganglion, composed of γ‐aminobutyric acid neurons, glutamatergic neurons, cholinergic neurons, and orexigenic neurons. Previous studies have focused on the involvement of the basal forebrain in regulating reward, learning, movement, sleep–awakening, and other neurobiological behaviors, but its role in the regulation of general anesthesia has not been systematically elucidated. Therefore, the different neuronal subtypes in the basal forebrain and projection pathways in general anesthesia will be discussed in this paper. In this paper, we aim to determine and elaborate on the role of the basal forebrain in general anesthesia and the development of theoretical research and provide a new theory.

## Graphical abstract

General anesthesia has been widely used in clinical practice for many years, but its underlying mechanism remains unclear. The basal forebrain (BF) is a major component in the ascending reticular activation system, which plays a key role in anesthesia. On the one hand, BF is classified into the following anatomical structures: The medial septum (MS), the nucleus basalis magnocellularis (NBM), the ventral pallidum (VP), the substantia innominate (SI), the level of division of diagonal band (HDB), and the magnocellular preoptic nucleus (MCPO). On the other hand, BF is made up of four types of neurons: cholinergic neurons, γ‐aminobutyric acid neurons, glutamatergic neurons, and orexigenic neurons. However, different types of cells in BF and different substructures of BF play different roles in regulating anesthesia and the mechanism is still unclear. In this review, we focus mainly on the role of BF in general anesthesia. Thus, the investigations of the BF will contribute toward understanding the mechanism of general anesthesia.

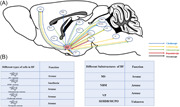

## 1 INTRODUCTION

General anesthesia has been widely used in clinical practice for many years, but its underlying mechanism remains unclear. One of the key issues in this field is determination of the trigger factors for awakening from general anesthesia. The basal forebrain (BF) is a major component in the ascending reticular activation system, which plays a key role in regulating locomotion, learning, and motivation,^1^ all of which rely on heightened arousal. Functional disorders of the BF are associated with neuropsychiatric disorders, including depression and drug addiction,^2^, ^3^ which are often accompanied by sleep disturbances, suggesting that the BF may play an important role in the regulation of sleep and wakefulness. In recent years, it has been found that the regulation of sleep‐wake cycles is similar to the loss of consciousness caused by general anesthesia. Specifically, general anesthetics act on the nucleus associated with the sleep–wake cycle, further contributing to the loss of consciousness. Indeed, many studies have been published about the relationship between BF and general anesthesia. Intravenous anesthetic propofol can reduce the calcium signal for neuronal activity in BF during induction.^4^ These studies showed that BF is an important transduction area for regulating loss of consciousness and recovery during general anesthesia.

## 2 ANATOMY OF THE BF

BF refers to a group of structures on the anterior and ventral sides of the cerebral hemisphere, including the anterior hypothalamus and the basal ganglia. BF is classified into the following anatomical structures: the medial septum (MS), the nucleus basalis magnocellularis (NBM), the ventral pallidum (VP), the substantia innominate (SI), the level of division of diagonal band (HDB), and the magnocellular preoptic nucleus (MCPO).^5^ BF is made up of four types of neurons: 5% cholinergic neurons (expressing choline acetyltransferase, ChAT+), 35% γ‐aminobutyric acid (GABA) neurons, 55% glutamatergic neurons, and 5% orexigenic neurons.^1^, ^6^ GABA neurons are further subdivided into parvalbumin‐positive (PV+) neurons and somatostatin‐positive (SOM+) neurons.^7^, ^8^ Glutamatergic neurons are further subdivided into those that express type 2 vesicular glutamate transporter neurons (Vglut2) and those that express type 3 vesicular glutamate transporter neurons (Vglut3).^9^

### 2.1 Intrinsic connections in BF

**Figure 1:**
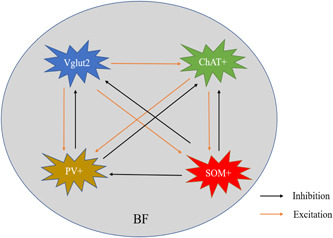
Schematic diagram of synaptic connections of various types of neurons in BF. BF, basal forebrain; ChAT+, choline acetyltransferase; PV+, parvalbumin‐positive; SOM+, somatostatin‐positive; Vglut2, vesicular glutamate transporter 2. [Color figure can be viewed at wileyonlinelibrary.com]

The distinct populations of neurons within the BF are interconnected. Using brain patch‐clamp techniques, the result showed that stimulation of Vglut2 neurons’ synaptic terminals in BF caused excitation of PV+ neurons, SOM+ neurons, and ChAT+ neurons. BF ChAT+ neurons excite PV+ neurons and SOM+neurons via nicotinic acetylcholine receptors. Activated SOM+ neurons significantly reduced firing of Vglut2 neurons, ChAT+ neurons, and PV+ neurons through GABA^A^ receptors. Specific optogenetics activation of PV+ neurons in BF generated spontaneous inhibitory currents in both Vglut2 neurons and ChAT+ neurons^10^, ^11^, ^12^, ^13^ (Figure 1).

### 2.2 Extrinsic connections out of BF

**Figure 2:**
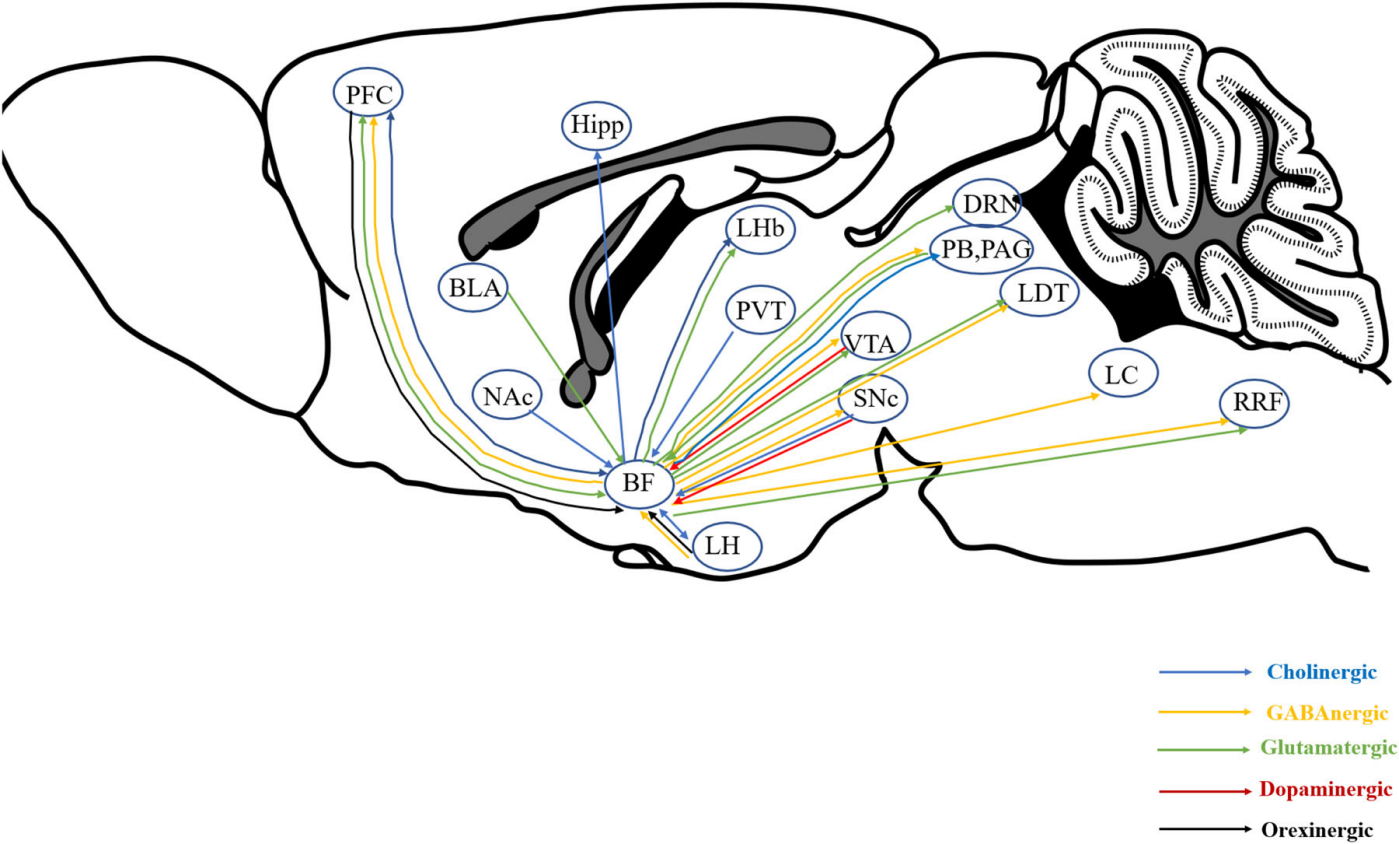
Projection between BF and nucleus related consciousness in the brain. BF, basal forebrain; BLA, basolateral amygdala; DRN, dorsal raphe nucleus; Hipp, hippocampus; LDT, laterodorsal tegmental nucleus; LC, locus coeruleus; LH, lateral hypothalamus; LHb, lateral habenula; NAc, nucleus accumbens; PAG, periaqueductal gray; PB, parabrachial; PFC, prefrontal cortex; PVT, paraventricular thalamus; RRF, retrorubral field; SNc, substantia negra; VTA, ventral tegmental area. [Color figure can be viewed at wileyonlinelibrary.com]

There are connections between BF and other brain areas related to consciousness. For instance, whole‐brain imaging reveals that the synaptic terminals of BF cholinergic neurons project to the hippocampus, the prefrontal cortex (PFC), the periaqueductal gray (PAG), the lateral habenula (LHb), and the lateral hypothalamus (LH).^14^, ^15^ GABA neurons in BF project to the ventral tegmental area (VTA),^16^ PFC, the substantia negra (SNc), the retrorubral field (RRF), the parabrachial (PB), the locus coeruleus (LC), the laterodorsal tegmental nucleus (LDT), the dorsal raphe nucleus (DRN), and PAG.^17^ Glutamatergic neurons in BF mainly project to VTA, RRF, DRN, LDT, LHb, LDT, and PFC.^18^ BF receives dense inputs from diverse brain regions. Cholinergic neurons in BF can be innervated by the nucleus accumbens (NAc), PFC, paraventricular thalamus (PVT), LH, VTA, and SNc.^19^ Furthermore, GABA neurons in LH also project to BF.^20^ Using anterograde transport autoradiography, Fallon discovered that BF receives dopaminergic neuronal input from the VTA and the SNc. Additionally, BF receives glutamatergic inputs from the PFC, PB, and basolateral amygdala,^21^ as well as orexinergic inputs from the PFC and LH^22^ (Figure 2).

## 3 THE ROLE OF BF IN GENERAL ANESTHESIA

### 3.1 Role of substructures of BF in general anesthesia

#### 3.1.1 MS

Each of the various subregions that make up the BF region is significant in the administration of general anesthesia. MS is a dorsomedial subnucleus located in the septal region's deep surface. MS, as part of BF, supports many physiological functions, from sensorimotor integration to cognition. Specifically, it has been reported that there is a link between MS and anesthesia. On the one hand, the movements induced by halothane, isoflurane, or propofol were significantly suppressed in MS‐inactivated rats compared with control rats. MS‐inactivated rats showed a prolonged loss of tail‐pinch and righting responses after administration of halothane or isoflurane. When administered intravenously at 10 mg/kg, propofol suppressed righting and tail‐pinch responses longer in MS‐inactivated rats.^23^ On the other hand, electrolytic lesions of MS prolonged the emergence from general anesthesia in rats. MS lesioned rats required a longer time to recover from a loss of righting reflex (LORR) after the administration of injectable or volatile anesthetic.^24^ MS damage alters not only the behavior of anesthetized mice but also the electroencephalogram (EEG) of anesthetized mice. One study found that hippocampal *γ* power in all rats decreased significantly in isoflurane, with lesioned rats with 192IgG‐saporin depleted cholinergic neurons in MS showing a greater decrease in *γ* ^2^ power than control rats under isoflurane anesthesia.^25^ This might be caused by loss of MS neurons, which disrupts the functional connectivity of the MS–hippocampus pathway. Another study^26^ explored the effects of MS glutamatergic transmission on hippocampal *θ* power via an MS microinjection of antagonist α‐amino‐3‐hydroxy‐5‐methyl‐4‐isoxazole‐propionic acid receptors (AMPARs). The results showed that microinjection of an AMPAR antagonist induced a decrease in the frequency of *θ* power in the anesthetized rat. Therefore, lesions of MS rats had a shorter induction time and a longer emergence time associated with anesthesia than sham rats.

#### 3.1.2 NBM

The NBM is found beneath the anterior commissure. Its significance in the arousal state regulation and anesthesia regulation has been reported. First, the anesthetic potency of propofol is potentiated because of the lesion of the NBM. In response to the pressure of the paw clamp, mice with NBM injury were more likely to not show the paw withdrawal reflex under propofol.^27^ This might be caused by 192IgG‐saporin depleting the choline in the NBM. In addition, the NBM has an impact not only on the process of awareness generated by intravenous anesthesia but also on the anesthetic effect of inhaled anesthetic medications. After 60 min of exposure to 2.1% inhaled anesthetic isoflurane, the time of recover the righting reflex (RORR) was significantly reduced in rats administered an infusion of histamine in the NBM,^28^ because histamine could depolarize NBM cholinergic cells mainly through histamine 1 receptors in NBM slices. Furthermore, the NBM also exerts an influence on the electroencephalographic power of mice administered inhaled anesthesia. For example, the average decrease in PFC *δ* power was 70% in rats administered desflurane anesthesia that responded after receiving norepinephrine microinfusion NBM. It seems that the mechanism of encephalographic arousal observed after norepinephrine microinfusion was mediated by corticofugal cholinergic modulation.^29^ Therefore, it can be seen that the NBM represents a principal anesthesia‐regulating region.

#### 3.1.3 VP

Under the anterior commissure, the VP is located. Based on its anatomy, the VP can be divided into two sections: the rostral ventral pallidum (RVP) and the caudal ventral pallidum (CVP). The neurons in the VP are typically oval, spindle shaped, or triangular in form, with a diameter of 15‐20 μm. Anesthesia exerts significant effects on the neural circuit function in the activated state. Optogenetic techniques with functional magnetic resonance imaging conducted in mice administered anesthesia have indicated that blood oxygenation level‐dependent activation in the VP accompanying optogenetic stimulation is strongly increased compared with that without optogenetic stimulation.^30^ Inactivation of the VP by muscimol infusion prolonged the duration of loss pain and righting responses under halothane.^31^ To investigate the mechanism of the role of the VP in anesthesia, Muller used microdialysis technology to measure the neurotransmitter content in the VP on administration of the intravenous anesthetic propofol. It was found that propofol decreases the release of dopamine in the VP via interacting with GABA^A^ receptors in the VP, without changing the glutamate transmitter level.^32^ However, as we all know, changes in the central nervous system's neurotransmitter composition are known to have an impact on anesthesia. For instance, an increase in GABA transmitters accelerates anesthesia, and increases in dopamine and glutamate transmitters promote arousal.^33^ These results confirm that the mechanism of propofol‐induced loss of consciousness is caused by a decrease in the content of excitatory transmitters like dopamine transmitters in the VP. Therefore, the VP serves as both a main target of propofol and a crucial area in the regulation of general anesthesia.

#### 3.1.4 HDB, MCPO, and SI

The HDB, MCPO, and SI, which are known as the HMS complex (HMSc), contain a large number of cholinergic neurons and project primarily to the LDT and the hippocampus.^34^ There is a strong link between the HMSc and the sleep–wake cycle. Initial examination of neuronal discharge patterns throughout the MCPO of the cat revealed that the most commonly encountered cell type did show peak discharge rates during waking and rapid eye movement sleep (REMS), with diminished discharge during nonrapid eye movement sleep (NREMS).^35^ In addition, neuronal firing in different states was also detected in rat MCPO. In the awake state, the discharge rate was closely related to movement. Compared with the active state, the discharge rate in the quiet state decreased significantly, and the discharge rate was the lowest in the deep NREMS state.^35^ Lesion of the SI led to an increase in slow waves of 1–4 Hz and an increase in REMS time.^36^ In the HDB, the spontaneous rate of warm‐sensitive neurons (WSNs) and cold‐sensitive neurons (CSNs) is recorded by 1–3 sleep–wake cycles. During waking and NREMS, the release of WSNs and CSNs is different. Of the 17 WSNs, 10 had an increased discharge rate during NREMS than during waking. Of the 20 CSNs, 14 CSNs discharged more slowly during NREMS than during waking.^37^ At present, the research on HMSc mainly focuses on the sleep–wake mechanism, and there is little research in the field of general anesthesia. Because the sleep–wake mechanism and the mechanism of loss of consciousness caused by general anesthesia have many common characteristics, the HMSc may play an important role in the mechanism of consciousness change caused by general anesthesia. Therefore, we need to further explore the role of HMSc in anesthesia.

### 3.2 Role of different types of neurons in BF in general anesthesia

#### 3.2.1 GABA neurons

GABA neurons were located throughout the BF. The highest density of GABA neurons was found in the MCPO region, followed by the HDB. GABA neurons were small (<15 μm long‐axis diameter), medium (15–20 μm long‐axis diameter), or large (>20 μm long‐axis diameter) sized. Large‐diameter neurons were more centralized in the SI, HDB, and MCPO. Medium‐diameter neurons were the highest in the HDB and the MCPO, whereas small‐diameter neurons were more centralized in the VP.^17^

In 2015, using an electrophysiological method, Jones found a relationship between the discharge patterns of GABA neurons and urethane anesthesia. There are two types of discharge patterns of GABA neurons under urethane anesthesia. One group presented a high frequency of discharge under deep urethane anesthesia. Another group showed a low frequency of discharge under light urethane anesthesia.^38^ This might indicate that there are two types neurons of GABA neurons that regulate anesthesia. Actually, GABA neurons in the BF can be classified into two types: One group includes PV + neurons and the other group includes SOM + neurons. In addition, they play different roles in regulating anesthesia. Through specific manipulation of the two types of neurons in general anesthesia, Yu and colleagues^39^ found that the SOM + neuron‐activated mice took less time to be anesthetized and longer time to recovery consciousness from isoflurane anesthesia. However, PV + neuron‐activated mice showed the reverse effect. In PV + neuron‐activated mice, the LORR time was longer and the RORR time was shorter under isoflurane anesthesia.^39^ This phenomenon may be attributed to the existence of neuronal circuits in BF. Activation of SOM + neurons can inhibit the firing of other excitatory types of neurons, resulting in silencing of neurons in BF. Hence, activation of SOM + neurons can increase the sensitivity of mice to anesthetic drugs.^10^, ^39^

#### 3.2.2 Glutamatergic neurons

Glutamatergic neurons are 15‐20 μm in diameter. The number of glutamatergic neurons in SI is the largest, followed by HDB, and the number of glutamatergic neurons in VP is the fewest.^9^ Interestingly, there is a mutual influence between propofol and glutamatergic neurons. On the one hand, propofol changes the firing of glutamatergic neurons in BF. On the other hand, changes in glutamatergic neuron activity can also affect the propofol anesthesia process. Previous studies have reported that propofol decreases the frequency and amplitude of spontaneous inhibitory postsynaptic currents of glutamatergic neurons in the BF through the GABA^A^ receptor.^40^ It is indicated that propofol can significantly inhibit the excitability of glutamatergic neurons in the BF through the tonic inhibition mediated by GABA receptors. To determine how to modulate the propofol process by changing glutamatergic neuron activity, local field potentials and cortical EEG were used to analyze the relationship between glutamatergic neuron and propofol. Wang found that cortical EEG and field potential during propofol anesthesia changed from a low‐frequency, high‐amplitude slow‐oscillating pattern to an active high‐frequency, low‐amplitude pattern as a result of glutamatergic neuron activation in the BF.^41^ All of these transmitter and electrophysiological studies suggest that glutamatergic neurons in BF play a role in the regulation of anesthesia.

#### 3.2.3 Cholinergic neurons

Cholinergic neurons in the BF have a maximal cell body size of 20 μm compared to noncholinergic neurons. Firing of cholinergic neurons yields distinct properties. In comparison to noncholinergic neurons, they have a lower resting membrane potential and a frequency of action potentials elicited by external depolarizing current stimulation.^42^ Cholinergic neurons are mainly distributed in HDB, followed by MCPO.^43^

Despite being few in number, cholinergic neurons in the BF have been the subject of the majority of research on how general anesthesia affects changes in awareness. First, at the cell level, propofol considerably raises energy barriers, the absolute refractory period of cells in the BF through GABA^A^ receptors.^44^ It is revealed that GABA^A^ receptors on cholinergic neurons allow propofol to drastically reduce the intrinsic excitability of cholinergic neurons in the BF. Second, in terms of behavior, Luo discovered that isoflurane and propofol had significantly greater anesthetic potency in mice in the presence of cell‐specific injury to BF cholinergic neurons and led to faster induction or longer emergence times.^45^ Finally, from EEG studies, it was found that selective cholinergic neuron activation using optogenetics in the BF markedly reduced low‐frequency power and increased power at 12–20 and 20–30 Hz during propofol anesthesia.^41^ Mice with vesicular acetylcholine transporter knockout in BF were compared with wild‐type mice. It was shown that the mice with reduction in this transporter in the BF showed a marked decrease in the strength of the *γ* wave under isoflurane anesthesia.^46^ Thus, all animal studies and electrical tests show that the cholinergic system in the BF regulates the process of general anesthesia.

#### 3.2.4 Orexigenic neurons

The number of orexigenic neurons in different subregions of the BF is varied. The number of orexigenic neurons in the SI is the largest, followed by the MCPO, and the number in MS is the fewest.^43^ Even though there are fewer orexigenic neurons in the BF, they are still important for regulating awareness during general anesthesia. The expression of the immediate early gene product c‐Fos has traditionally been considered to be an indicator of transcriptional activation. After isoflurane anesthesia, the number of c‐Fos‐positive orexigenic neurons in the BF decreased significantly, but increased significantly after anesthesia termination and reflex recovery.^47^ However, the current study has only shown that orexin neurons in the BF can be affected by general anesthetics; the precise process is still unknown.

## 4 ROLE OF PROJECTION PATHWAYS ASSOCIATED WITH THE BF IN GENERAL ANESTHESIA

### 4.1 NAc‐BF GABAergic pathway

Using anterograde and retrograde tracers and electrophysiological methods, Clarke confirmed that the main input of VP are innervations from NAc.^48^ More than 80% of GABA neurons in the NAc project to the VP, and there is an inhibitory projection from the NAc synaptic cells to the VP GABAergic synaptic cells.^49^ Hemmings discovered that blocking the NAc–VP pathway prolonged mice's recovery from halothane or pentobarbital anesthesia and increased the sensitivity to general anesthesia.^50^ This suggests that the NAc–VP pathway is involved in the regulation of the emergence process of general anesthesia.

### 4.2 BF‐PFC cholinergic pathway

Cholinergic neurons in the BF are the primary source of acetylcholine transmitters in the PFC. Changes in the BF's functionality of cholinergic neurons may have an indirect impact on the input of glutamatergic and GABA neurons to the cerebral cortex.^51^ As a result, the BF–PFC cholinergic pathway plays a role in how the effects of general anesthesia on consciousness are regulated in both direct and indirect ways. Greater BF cholinergic neuron activity results in increased cortical acetylcholine transmitter release, which stimulates the cortex and decreases sensitivity to anesthetic medications.^52^ The PFC‐BF pathway still regulates general anesthesia. Electrical stimulation of BF during sevoflurane anesthesia resulted in high‐frequency power in mice, but tetrodotoxin injection into the PFC reduced this effect.^52^ A neuronal circuit between the BF and the PFC regulates the process of loss of consciousness brought on by general anesthesia. The PFC's transmitters can be altered by the BF to increase arousal. Decreased neuronal activity in the PFC can reduce the pro‐arousal effect after BF activation.

### 4.3 LH–BF orexinergic pathway

There are numerous orexinergic neurons in the perifornical lateral hypothalamic (PeFLH), and their synaptic terminals can extend into the BF. The BF regulates emergence from propofol and sevoflurane anesthesia via orexin modulation.^47^ Under 1.4 vol% isoflurane anesthesia, compared with control groups, the burst suppression ratio was less and emergence time was shorter in groups with optogenetic activation of orexinergic cell bodies in the PeFLH.^22^ Therefore, optical stimulation of cell bodies of orexinergic neurons in the PeFLH promotes arousal from anesthesia. In addition, photoactivation of orexinergic terminals in the BF caused a significant decline in the burst suppression ratio.^22^ It is possible that activation of the orexin terminal of the LH in the BF results in greater release of orexin transmitters and promotes recovery from anesthesia in mice.

### 4.4 PB–BF glutamatergic pathway

The PB of the dorsolateral pons provides extensive, largely glutamatergic neurons, ascending innervation of BF, and descending projections to medullary regions. ChR2 virus was injected into PB and we found that an increased firing frequency and excitatory postsynaptic current (EPSC) were elicited by blue light pulses in BF.^21^ This shows that the PB and the BF are connected by a neuronal circuit and functional alterations of neurons in the BF, the downstream nuclear, can be regulated by the PB, the upstream nuclear. In addition, hM3Dq virus was injected in PB of mice and treated with vehicle or Clozapine N‐oxide (CNO), Huang found CNO injection markedly rose the number of c‐Fos–positive neurons in BF by 2.8‐fold compared with vehicle during sevoflurane anesthesia.^53^ This indicates that excitation of PB glutamatergic neurons increases activation of BF, which may speed up the transition from general anesthesia to an arousal state.

## 5 SUMMARY AND OUTLOOK

The BF is a crucial nucleus in the brain's ascending reticular system. Although the BF is closely related to other brain regions, it is believed that there are intricate microcircuits between different types of BF neurons. Different neuronal types collaborate to regulate the development of anesthesia–arousal responses. Currently, there is some understanding of the function of cholinergic, GABA, glutamatergic, and orexinergic neurons in the BF during anesthesia. Additionally, the neural pathways that the BF projects to nuclear areas like the PFC and NAc are involved in regulating general anesthesia. The BF is a very complex system. This paper only describes some of the research results, but provides a direction for future research and development. It is hoped that the relationship between brain function, consciousness formation, and anesthesia will become more obvious with continuous advancements of brain science and technology, such as optogenetics and transmitter probe technologies.

## AUTHOR CONTRIBUTIONS

Yi‐Ting Peng and Yi Zhang contributed to the main conception of the study and resource collection; Cheng‐Dong Yuan contributed to drafting and editing of the manuscript; and Yi Zhang finalized the review and approved the final version.

## CONFLICT OF INTEREST

The authors declare no conflict of interest.

## ETHICS STATEMENT

The ethics statement is not available.

## TRANSPARENCY STATEMENT

The authors affirm that this manuscript is an honest, accurate, and transparent account of the study being reported and that no important aspects of the study have been omitted.

## Data Availability

Data sharing is not applicable to this article as no data sets were generated during the current study.
